# Intracerebral Hemorrhage: The Effects of Aging on Brain Injury

**DOI:** 10.3389/fnagi.2022.859067

**Published:** 2022-04-25

**Authors:** Noah Watson, Frederick Bonsack, Sangeetha Sukumari-Ramesh

**Affiliations:** Department of Pharmacology and Toxicology, Medical College of Georgia, Augusta University, Augusta, GA, United States

**Keywords:** intracerebral hemorrhage, aging, microglia, neuroinflammation, iron

## Abstract

Intracerebral hemorrhage (ICH) is a devastating subtype of stroke with high rates of mortality and morbidity. ICH patients often suffer devastating and debilitating neurological impairments, from which the majority of victims are unable to fully recover to functional independence. Unfortunately, there is no established medical therapy for ICH, which is partly attributed to the lack of understanding of the complex pathology of the disorder. Despite advanced age being a major risk factor of ICH, most preclinical studies on ICH employed young animal subjects. Due to this discrepancy, the molecular level changes in the aging brain after ICH are largely unknown, limiting the translation of preclinical studies into potential human treatments. The purpose of this review is to highlight the effects of advanced age on ICH- induced brain injury and recovery and to draw attention to current knowledge gaps, which warrant further investigation.

## Introduction

Intracerebral hemorrhage (ICH) is the second most common form of stroke, caused by blood vessel rupture and subsequent bleeding into the surrounding brain tissue (Qureshi et al., 2009). ICH accounts for 10–20% of stroke cases worldwide (Feigin et al., 2009; Sacco et al., 2009), with incidence varying across different countries and ethnicities. For instance, its prevalence is much higher in low-middle income countries, which have a higher proportion of fatal cases (Feigin et al., 2009). The incidence of ICH in African Americans is twice as high compared to white Americans (Flaherty et al., 2005). Notably, the worldwide incidence of ICH has risen by ∼47% over the last 20 years (An et al., 2017), and hospital admissions have increased by 18% in the past 10 years (Qureshi et al., 2007). Moreover, the United States population is aging at an unprecedented pace and the fastest-growing age group in the United States is those over the age of 65 (Wasil and Lichtman, 2005). It is projected that by 2030, 20% of the United States population will be over the age of 65, compared to 2010, when this demographic only accounted for 13% of the population (Albright et al., 2016). As the elderly population continues to grow, the prevalence of ICH could rise alongside it since advanced age is a major risk factor of ICH. By 2030, nearly 4% of the United States population is estimated to have had a stroke (Ovbiagele et al., 2013).

Intracerebral hemorrhage imposes a significant economic burden on society, contributing to an estimated $17.2 billion in annual direct costs to the U.S. healthcare system associated with stroke (Taylor et al., 1996; Caro and Huybrechts, 1999; Reed et al., 2001; Mozaffarian et al., 2015). The 30-day mortality rate of patients suffering from ICH is high, about 40%, with only around 20% of survivors achieving functional independence 6 months after the onset of brain hemorrhage (Bonsack and Alleyne, 2016). A critical barrier to improve patient outcomes after ICH is the lack of effective treatment, partly attributed to the complexity and the poorly defined pathophysiology of the disorder. Though surgical approaches are offered for selected patients, elderly patients, which comprise approximately one-third of the ICH population, are less likely to undergo surgical treatment, perhaps due to established end-of-life preferences. Taken together, the current treatment option for ICH, even in dedicated stroke centers, is limited to supportive care.

The risk factors of ICH include hypertension, cerebral amyloid angiopathy, advanced age, cigarette smoking, diabetes, alcohol abuse, drug abuse, Asian ethnicity, genetic factors, menopause, and oral anticoagulant treatment (Feldmann et al., 2005; Morotti and Goldstein, 2016). Also, there was a significant association between lower LDL-C (Low-Density Lipoprotein-Cholesterol; <70 mg/dL) and higher risk of ICH (Ma et al., 2019). However, a recent meta-analysis reported that lipid-lowering therapy was not associated with a significantly increased risk of ICH in primary prevention trials (Judge et al., 2019), while a more recent meta-analysis concluded that lipid-lowering therapy increased the risk of ICH, but only at high doses that achieved ≥35% reduction in LDL-cholesterol levels (Cheng et al., 2020). Due to these discrepancies, further studies are highly required to determine the precise role of LDL-C in the pathogenesis of ICH. The most common risk factor of ICH is hypertension (Morotti and Goldstein, 2016), which accounts for approximately 83% of ICH cases (Cheung and Zou, 2003). Hypertension nearly doubles the risk of ICH (Jackson and Sudlow, 2006) and usually leads to ruptures of vessels at the bifurcation of small arteries within the brain (Qureshi et al., 2009). It is believed that long-standing elevated blood pressure promotes shear stress and degenerative changes to the walls of small-to-medium penetrating vessels causing vascular ruptures and ICH (Qureshi et al., 2009). Hypertension is also a key contributor to aneurysmal rupture leading to ICH (van Asch et al., 2010b; Jabbarli et al., 2016) and hypertensive ICH occurs mostly in deep brain structures (Matsukawa et al., 2012). Another risk factor that accounts for ∼20% of ICH cases is cerebral amyloid angiopathy (CAA), which is prevalent in elderly patients (Morotti and Goldstein, 2016; An et al., 2017). CAA is manifested by the deposition of amyloid-β peptides in small-to-medium-sized arteries and arterioles in the cortex and leptomeninges, resulting in lobar hemorrhage (Matsukawa et al., 2012). CAA accounts for approximately 12–15% of lobar ICH in the elderly (Itoh et al., 1993; Mehndiratta et al., 2012), and the risk of developing sporadic CAA-related ICH increases in carriers of the ε4 and ε2 alleles of apolipoprotein E, the monogenic risk factor of ICH (Nicoll et al., 1997; Sudlow et al., 2006; Talha et al., 2020).

As a person gets older, the risk of developing ICH increases. Elderly individuals have a fivefold higher risk of ICH as opposed to their younger counterparts (van Asch et al., 2010a). Age enhances the risk of chronic health conditions and systemic conditions such as hypertension, diabetes and atrial fibrillation (Ariesen et al., 2003), which can contribute to the pathophysiology of ICH. Elderly patients make up a significant portion of the ICH population, and as per a recent study ∼34% of ICH patients were 80 years or older (Stein et al., 2012). Studies have shown a steady increase of ICH cases per 100,000 individuals from 5.9 in 35–54 year-olds to 176.3 in 75–94 year-olds (Wasil and Lichtman, 2005). Age increases not only the prevalence of ICH exponentially (Broderick et al., 1993), but also the 30-day mortality rate (Gonzalez-Perez et al., 2013). The mortality rate in men raised from 23% in ICH patients under 75 years of age to 41% in those over 75 (Zia et al., 2009). Furthermore, age is an independent predictor of poor functional outcomes when measured *via* total Functional Independence Measure (FIM) score and Motor FIM score (Bagg et al., 2002) and age (>65 years) is an independent predictor for recurrent ICH (Zia et al., 2009). The sex differences in outcomes have not been fully characterized in the pathophysiology of ICH. For younger patients, female sex was protective, but at age >60 years, female sex was a risk factor for death or discharge to hospice (Umeano et al., 2013; Craen et al., 2019). The mortality rate of ICH increased from 20% in female patients under 75 to 26% in those over 75 (Zia et al., 2009). However, the role of aging in the pathophysiology of ICH remains largely understudied. The lack of preclinical studies limits our understanding of the intricate molecular mechanisms of ICH-induced brain injury and the translation of preclinical studies into potential human treatments. Although preclinical animal models of ICH are potent tools for characterizing the disease pathology, most ICH research has employed young animal subjects. This discrepancy may be in part due to the increased amount of time and cost that need to be invested for aging-related studies and the limited commercial availability of aged animals. Given that the elderly population accounts for approximately one-third of ICH patients coupled with the possible increase in prevalence of ICH, herein we provide an overview of the multifactorial effects of aging in the pathophysiology of ICH and identify the knowledge gap, which could help develop new research avenues to improve the prognosis of ICH patients.

### Cerebrovascular Circulation and Intracerebral Hemorrhage

Aging is an intricate phenomenon and there are numerous effects of aging on the body. Age-related changes in the cerebral vasculature include vascular stiffness, decreased vascular density, thickening of the vessel wall, endothelial dysfunction, and increased blood-brain barrier permeability (Xu et al., 2017). Overall, these age-induced changes to the vasculature can make the brain parenchyma more susceptible to ICH-induced brain damage apart from enhancing the risk of ICH. Consistently, aged rats (18-months) exhibited significantly higher neurobehavioral deficits after ICH than young rats (3-months) and this was coupled with augmented brain edema in the aged group at 3 days post-ICH (Gong et al., 2005). Additionally, residual brain lesion volume was significantly enhanced in the aged rats at 28-days post-ICH compared to their younger counterparts, suggesting aging-associated impairment in lesion resolution (Wasserman et al., 2008). However, it is largely unknown how aging precisely modulates neurological deficits, cerebral edema and lesion resolution after ICH, warranting investigation.

Age increases the prevalence of hypertension (Buford, 2016) and CAA (Love et al., 2003), the common causatives of ICH. Hypertension in the context of aging contributes to arterial stiffening and remodeling (Sun, 2015), factors that could predispose to ICH. The vasculopathic changes that are often associated with CAA include loss of smooth muscle cells, vessel wall thickening, lumenal narrowing, concentric splitting of the vessel wall and microaneurysm formation (Viswanathan and Greenberg, 2011). In addition, lobar cerebral microbleeds (CMB or cerebral microhemorrhages: CMH), small focal intracerebral hemorrhages and key contributors of cognitive decline and dementia in older adults (Akoudad et al., 2015), are often found in conjunction with CAA (Viswanathan and Greenberg, 2011). However, the mechanism of blood vessel rupture in CAA is yet to be defined. It is assumed that replacement of the smooth muscle cells of the media by amyloid deposits result in vessel wall weakening and subsequently, vascular rupture (Winkler et al., 2001). It remains largely unclear why some CAA-associated vessel ruptures result in ICH, while others culminate in microhemorrhages. Though the presence of cerebral microbleeds is not significantly associated with the risk of ICH or clinical outcome (Derraz et al., 2021), microbleeds may serve as predictors of ICH recurrence (Tsushima et al., 2003; Greenberg et al., 2004).

Hypertension is another risk factor for CMHs (Vernooij et al., 2008) and aging promoted hypertension induced-cerebral microhemorrhages in a mouse model that recapitulated cerebromicrovascular alterations in elderly humans (Toth et al., 2015). Mechanistically, age-induced reduction in IGF-1 (insulin-like growth factor -1) signaling and reactive oxygen species-mediated activation of MMPs (Matrix Metalloproteinases) in the cerebrovasculature could make the cerebral blood vessels more vulnerable to hypertension-induced rupture (Tarantini et al., 2017). However, despite the association between increased MMP-9 activation and the genesis of ICH in various experimental murine models (Lee et al., 2003, 2007), genetic deletion of MMP-9 did not attenuate neurological manifestations associated with hypertension-induced ICH in aged mice (Tarantini et al., 2021). Though this is an important observation, there is a possibility that in MMP-9 null mice, other MMP isoforms could express in dysregulated manner and overcompensate the effects of MMP-9 deletion (Tang et al., 2004) and hence, further studies are required with selective pharmacological agents to determine the role of MMP-9 in the pathogenesis of ICH.

Despite an emerging interest in elucidating the association between cerebral microbleeds and ICH, the mechanisms of the development of cerebral microbleeds are largely obscure and complex. A recent study documented that induction of severe systolic hypertension in mice could alter the neurovascular unit resulting in microhemorrhages in the brain (de Montgolfier et al., 2019). Moreover, cerebral venous congestion can contribute to brain microhemorrhages in mice (Nyul-Toth et al., 2022), implicating a novel role of venous circulation in the genesis of cerebral microbleeds, requiring further studies.

### Immune Response and Intracerebral Hemorrhage

Aging is a complex process and the immune system experiences significant changes with advanced age. Consistently, in the healthy aged brain, the augmented activation of microglia, the cells that play critical roles in innate immune response, has been reported in diverse mammalian species, including humans (Peters et al., 1991; Dickson et al., 1992; Ogura et al., 1994; Sheffield and Berman, 1998). Furthermore, advanced age is associated with neuronal death, a decline in cognitive function (Ginaldi et al., 1999; Rawji et al., 2016), and a chronic low-grade inflammatory state known as inflamm-aging that is characterized by elevated levels of proinflammatory cytokines (Gabuzda and Yankner, 2013). It is believed that senescence of immune cells and age-dependent changes in macromolecules contribute to inflamm-aging, which, in turn, could partly be responsible for the impaired innate and adaptive immune responses seen in the elderly (Deleidi et al., 2015; Frasca and Blomberg, 2016).

Similar to other bodily tissues, a direct injury to the brain will result in the rapid release of local inflammatory factors and the recruitment of immune cells (Carson et al., 2006). Consequently, microglia undergo alterations in phenotypic, phagocytic, and antigen presentation properties (Sheffield and Berman, 1998). The activated microglia are regarded as the key cellular regulators for neuroinflammation following ICH, owing to their ability to secrete cytokines, chemokines, reactive oxygen species, and prostaglandins (Aronowski and Hall, 2005; Wang and Dore, 2007). The release of these factors further exacerbates microglial activation and recruit blood-derived monocytes/macrophages into the brain, together modulating the inflammatory response (Tessier et al., 1997; Melton et al., 2003; Nakanishi, 2003; Shiratori et al., 2010; Starossom et al., 2012; Chang et al., 2017) and contributing to ICH-induced brain injury (Platt et al., 1998; Hickenbottom et al., 1999; Leira et al., 2004; Zhao et al., 2007). ICH results in the release of a cascade of stimuli that activate microglia/macrophages, which include blood components such as thrombin, hemoglobin, plasma proteins, and hemoglobin degradation products such as hemin and iron (Bonsack and Alleyne, 2016). Several of these factors interact with a class of pattern recognition receptors, Toll-like receptors (TLRs), located on microglia/macrophages and activate proinflammatory signaling such as NFkB or NLRP3 (Dasari et al., 2021). Along these lines, TLR-4 is a key regulator of inflammatory brain damage after ICH (Lin et al., 2012; Wang et al., 2013). Notably, the proinflammatory activation of microglia/macrophage after ICH correlates with blood-brain barrier damage, brain swelling/edema, hematoma expansion, neurological deterioration, and poor functional recovery (Platt et al., 1998; Hickenbottom et al., 1999; Leira et al., 2004; Zhao et al., 2007), implicating microglia as a key contributor of ICH-induced secondary brain injury and loss of neurological function.

Though microglia/macrophage characterization after ICH has primarily been carried out in young animal subjects, it is reported that the number of activated microglia/macrophages is significantly increased in elderly rats after ICH compared to younger rats (Gong et al., 2004) in line with severe brain injury observed in aged rats. Furthermore, microglia exhibited widespread activation in the ipsilateral brain parenchyma in aged rats after ICH (Wasserman et al., 2008). However, it is largely understudied whether and how aging orchestrates the microglial release of various inflammatory mediators and brain injury after ICH. Studies carried out in young mice have shown that microglia/macrophages undergo polarization after ICH and exhibit pro-inflammatory M1 phenotype or anti-inflammatory M2 phenotype (Bonsack and Alleyne, 2016). The classical activation of microglia/macrophage that gears toward M1 phenotype releases proinflammatory cytokines IL-1β, IL-6, IL-8, and TNF-α and reactive oxygen species, thereby contributing to brain damage (Wan et al., 2016; Zhang et al., 2016; Lan et al., 2017). In contrast, an alternate activation of microglia yields an anti-inflammatory M2 phenotype, releasing anti-inflammatory cytokines such as IL-10, IL-4, IL-13, and transforming growth factor β (TGFβ), culminating in brain recovery (Ni et al., 2016). In line with the detrimental and beneficial role of M1 and M2 microglia/macrophage, respectively, a reduction of M1 or an increase of M2 microglia/macrophage was associated with neuroprotection in the acute phase of ICH. However, studies are yet to be conducted to elucidate how aging alters classical or alternate activation of microglia after ICH and whether microglial phenotypes are viable targets to improve outcomes after ICH in the elderly (Spittau, 2017).

Microglia themselves show age-related changes in phenotype and functionality. Aged microglia are described as *dystrophic or* senescent (Candlish and Hefendehl, 2021), which exhibit many phenotypic changes compared to young microglia, such as increased soma volume and less arborization, meaning fewer and shorter processes (Koellhoffer et al., 2017; Spittau, 2017). *Dystrophic* microglia, to some extent, are comparable to activated microglia. Functionally, these dystrophic microglia show reduced chemotaxis and process motility, suggesting that they could respond differentially to neuropathology (Spittau, 2017). Moreover, the number of *dystrophic* microglia significantly increases as individuals age, especially in people with neurodegenerative diseases (Shahidehpour et al., 2021). A potential reason for the increased activation of microglia in the aged brain could be aging-induced myelin breakdown and subsequent activation of the microglia in response to the changes in the brain microenvironment and as an attempt to engulf myelin debris (Conde and Streit, 2006). In addition, studies have shown that aging could shift microglia to a constant low-grade inflammatory state (Pan et al., 2020; Candlish and Hefendehl, 2021), suggesting that microglia could play a critical role in “inflamm-aging,” which is partly responsible for age-associated impairments such as decreased remyelination, memory deficits, and gray matter loss (Koellhoffer et al., 2017). Furthermore, aging could prime microglia to a proinflammatory M1 phenotype. Consistently, microglia in aged rats exhibited increased expression of MHC II (Henry et al., 2009), a marker of M1 microglial phenotype. Moreover, aged microglia were associated with enhanced mRNA expression of proinflammatory cytokines such as TNFα, IL-1β, and IL-6 as well as anti-inflammatory cytokines, IL-10 and TGFβ1 (Sierra et al., 2007). Moreover, mixed glial cultures from aged mice produced elevated levels of proinflammatory cytokines upon lipopolysaccharide (LPS) treatment compared to those established from young adult mice. Also, microglia from aged mice retained a classically activated or M1 phenotype in the presence of IL-4 (Fenn et al., 2012). In contrast, microglia from young adult mice were responsive to anti-inflammatory cytokine, IL-4 and its treatment shifted microglial phenotype toward an alternatively activated M2 (Fenn et al., 2012). Overall, an enhanced response to proinflammatory signals coupled with a reduced microglial sensitivity to IL-4 could result in exaggerated and prolonged neuroinflammation, amplifying neurodegeneration in the aging brain upon a brain injury. Consistently, it was demonstrated that the expression of IL-1β protein after ICH was greater in aged rats than in young rats (Lee et al., 2009). Altogether, the age-induced alterations in inflammatory microglial responses could contribute to ICH-induced brain injury and the disproportionate deficits and recovery rates in older patients.

Intracerebral hemorrhage results in both primary and secondary brain injury. The primary brain injury results from the development and mass effect of the hematoma. In contrast, the secondary brain injury, which persists for an extended period and often results in long-term neurological deficits, involves a multitude of mechanisms mostly induced by hematoma components, such as neuroinflammation, oxidative brain damage, and blood-brain damage. Importantly, the volume of the initial hematoma correlates with morbidity and mortality following ICH, and hematoma expansion was associated with poor patient prognosis (Fujii et al., 1994). Altogether, the timely removal of hematoma, the ongoing source of brain damage, is critical for brain recovery after ICH. To this end, apart from the role of microglia in inflammatory brain responses after ICH, studies document that microglia and brain infiltrating macrophages could regulate hematoma resolution and brain recovery owing to their ability to phagocytose cellular debris that accumulates in the brain after a brain injury. Moreover, phagocytosis or removal of dying cells is necessary to prevent the release of intracellular inflammatory agents such as damage-associated molecular patterns (DAMPs) (Sims et al., 2010). Therefore, identification and characterization of endogenous molecular regulators of microglial or macrophage-mediated phagocytosis could improve outcomes after ICH. Of note, elderly subjects with ICH had a larger hematoma volume with poorer outcomes than younger patients (Inoue et al., 2018) partly due to age-mediated parenchymal degeneration and subsequent reduction in the structural integrity of the brain tissue, which could otherwise restrict hematoma growth. Moreover, aged microglia exhibited reduced expression of genes associated with phagocytosis (Orre et al., 2014) and TGFβ-induced phagocytosis was abolished in aged microglia compared to their younger counterparts (Tichauer et al., 2014). Also, aging can enhance the infiltration of brain-infiltrating monocyte-derived macrophage (macrophage/BMDM) after a brain injury (Chou et al., 2018) and modulate its responses, such as the release of inflammatory mediators and phagocytosis (Albright et al., 2016; Rawji et al., 2016). However, how aging functionally alters microglial or BMDM-mediated inflammatory responses, phagocytosis, and hematoma resolution after ICH remains enigmatic, warranting studies.

Another hallmark of brain aging is increased oxidative stress and lipid peroxidation. A prevailing hypothesis is that the age-induced accumulation of free radical damage promotes neuroinflammation. Consistently, there was an overall increase in pro-oxidant and inflammatory genes, while there is a reduction in anti-oxidant genes in the brain of older rodents compared to adults (Lee et al., 1999; Godbout et al., 2005). Furthermore, reactive oxygen species could drive persistent microglial activation (Qin et al., 2013) and promoted M1 microglial activation (Taetzsch et al., 2015). Also, cells damaged by oxidative stress could produce inflammatory factors (Shao et al., 2020), further implicating a role of oxidative stress in neuroimmune responses, warranting investigation. Moreover, age-mediated alterations in the levels of circulating factors such as cytokines could regulate brain injury (Huang et al., 2020). Along these lines, plasma from young rodents could alleviate acute brain injury post-ICH in aged rodents (Yuan et al., 2019), lending support to the conclusion that circulating factors contribute to neural deficits and increased injury after ICH in the elderly.

### Iron and Intracerebral Hemorrhage

Iron is a key contributor to both acute as well as delayed brain damage after ICH (Nakamura et al., 2003). The brain concentration of iron, a hemoglobin degradation product, reaches very high levels post-ICH due to erythrocyte lysis and subsequent release of hemoglobin into the extracellular space. A threefold increase of brain non-heme iron after intracerebral hemorrhage was observed in rats (Wu et al., 2003). Iron accumulation in the brain triggers a cascade of deleterious reactions such as free radical production, mitochondria damage, and macrophage/microglial activation, disrupting cellular homeostasis and culminating in neuronal death, oxidative and inflammatory brain injury, and neurological deficits after ICH (Dai et al., 2019). In the acute phase of ICH, hemolysis-generated iron can potentiate thrombin-induced neurotoxicity (Nakamura et al., 2005) and contribute to cerebral edema (Xi et al., 2002b). Although the molecular mechanisms of iron-induced neurotoxicity are not fully understood, iron levels in the brain remain high for at least several weeks post-ICH (Wu et al., 2003), which could contribute to long-term neurological deficits. Importantly, aging is often associated with excess iron accumulation in the substantia nigra, putamen, globus pallidus, caudate nucleus, and cortices (Zecca et al., 2004; Ramos et al., 2014; Ward et al., 2014), which could further modulate brain damage after ICH. In addition, age-mediated enhancement in erythrocyte fragility (Orbach et al., 2017), may alter the rate of erythrocyte lysis subsequent to ICH, resulting in increased heme or iron-induced brain damage. Consistently, the level of the iron-regulatory protein, heme-oxygenase 1, was elevated in the aged rat after ICH compared to young rats (Gong et al., 2004). Of note, genetic overexpression of ferroportin 1, an iron exporter, led to less iron accumulation, less neuronal apoptosis, and improved neurological outcomes in aged mice (Bao et al., 2020), further implicating a role of iron in ICH pathophysiology.

Evidence has been shown that advanced age is associated with enhanced complement activation (Gong et al., 2008), which plays a role in the formation of membrane attack complexes (MAC) (Hua et al., 2000; Ducruet et al., 2009), resulting in erythrocyte lysis and hence, hemoglobin or iron-mediated neurotoxicity (Yuan et al., 2019) and cerebral edema development after ICH (Xi et al., 2001, 2002a; Yang et al., 2006a,b). Moreover, complement components such as C3a anaphylatoxin could also contribute to ICH pathology by enhancing vascular permeability (Foreman et al., 1996) and leukocyte infiltration. Consistent with the role of complement activation in brain injury, intracerebral administration of a complement inhibitor reduced erythrolysis, iron accumulation, microglial activation, cerebral edema, and neuronal death in aged rats after ICH (Yuan et al., 2019). Furthermore, complement components may play a role in the clearance of apoptotic cell bodies and contribute to ischemic stroke-induced neurogenesis (Rahpeymai et al., 2006), implicating its unexplored role in brain recovery after ICH. Therefore, further studies are required to determine the precise molecular mechanisms by which complement activation modulate brain damage or recovery and whether systemic administration of a complement inhibitor is a feasible strategy to improve neurological outcomes in aged mice after ICH.

White matter injury is a frequent complication of ICH (Tao et al., 2017) and as per a report, more than 77% of ICH patients suffered white matter injury (Smith et al., 2004). White matter injury is observed in both acute and chronic phases of ICH and is characterized by demyelination, axonal damage and oligodendrocyte death (Ni et al., 2015). Though the precise mechanism of white matter injury after ICH is enigmatic, iron-induced oxidative stress could culminate in white matter damage (Li et al., 2021). In a rat model of ICH, white matter injury correlated with brain edema and poor neurological outcomes (Tao et al., 2016). Moreover, white matter injury is a major cause of sensory-motor deficits commonly seen in ICH patients (Li et al., 2021) and was associated with cognitive impairment (Smith et al., 2004). Of note, aging is often associated with cerebral white matter lesions characterized by demyelination, gliosis, and capillary degeneration (Hoffman et al., 1985; Baltan et al., 2008; Asdaghi et al., 2012). Also, aging can augment white matter vulnerability to ICH-induced brain damage. Altogether, additional studies are required to delineate the age-induced changes in iron metabolism and molecular mechanisms of iron-induced neurological deficits in the aging population after ICH.

### Cognition and Intracerebral Hemorrhage

There is a high prevalence of dementia after ICH (ranging from 9 to 29% for pre-ICH and 14–88% for post-ICH) (Donnellan and Werring, 2020) and dementia could be a predictor of mortality in ICH survivors (Judge et al., 2019). After ICH, cognitive deficits could arise from the acute hemorrhagic lesion or in a progressive manner owing to slowly accumulating vascular and non-vascular pathology (Xiong et al., 2016). Notably, cognitive impairment after ICH remains largely understudied. In a preclinical rodent model of ICH, no significant learning or memory deficits were observed 1–7 months post-ICH (MacLellan et al., 2009). However, in another study using the same model, there were significant learning deficits at 2 weeks post-ICH, but the learning deficits reduced remarkably at 8 weeks post-ICH (Hartman et al., 2009). These conflicting results warrant additional investigation. Moreover, these studies were conducted in young animal subjects, which lack underlying neuropathology, which could otherwise be needed for the development of cognitive impairment after ICH, apart from hemorrhage-induced brain damage. To this end, employing aged animal subjects could better establish the association between ICH and cognition, which may improve the prognosis of ICH survivors.

### Prognostic Factors and Intracerebral Hemorrhage

Given the devastatingly high morbidity and mortality associated with ICH, the predictors of patient prognosis carry high clinical significance. The predictors of adverse patient outcome include advanced age, enhanced ICH volume, presence of intraventricular hemorrhage, low Glasgow Coma Scale score (GCS score) and deep/infratentorial ICH location (Poon et al., 2014). Evaluation of these prognostic factors helps establish an ICH score or a risk stratification scale predicting 30-day mortality (Hemphill et al., 2001). Of note, Yang et al. (2020) found that the predictors of patient mortality differ between young and aged ICH patients. To this end, brain herniation in the young group, and low GCS scores, renal or heart disease, and leukocytosis in the elderly were associated with higher 1-month mortality (Yang et al., 2020). Also, studies on elderly patients with age ≥75 years demonstrated that a hematoma volume ≥30 ml, or a prior history of ICH was associated with a higher likelihood of short-term death (Batista et al., 2021). Apart from these, blood-derived inflammation markers could serve as prognostic indicators. Along these lines, increased plasma level of TNF-α was associated with mortality in ICH patients (Fang et al., 2007). As per another study, elevated plasma level of IL-6 is an independent predictor for early hematoma growth, which, in turn, is associated with poor outcomes following ICH (Silva et al., 2005). Though inflammatory biomarkers that could predict better recovery after ICH are least characterized, as per a recent report, increased serum levels of IL-33, a newly identified member of the IL-1 family, were found in patients with improved functional outcomes compared to those with poor outcomes (Miao et al., 2021). It is important to highlight that these studies investigating potential ICH biomarkers are not limited to the elderly population, but often include any patient of adult age. Therefore, further studies are needed to determine whether these predictive markers are as effective when solely looking at elderly patients.

## Conclusion

**FIGURE 1:**
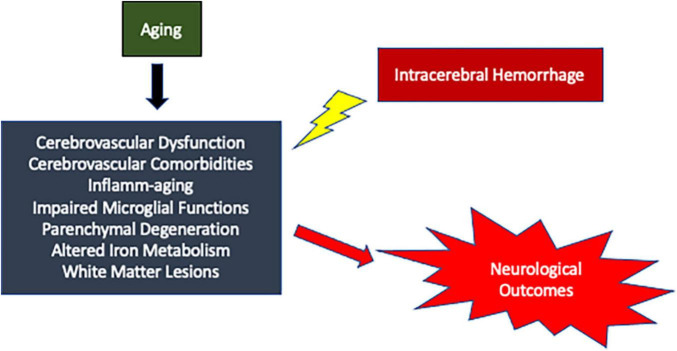
Schematic representation of possible mechanisms by which aging could modulate brain damage after ICH resulting in worse neurological outcomes.

Intracerebral hemorrhage is a complex disorder with no effective treatment. Aging has a multifaceted effect on the development and the progression of the disease (Figure 1). Therefore, aging could impose a myriad of unique challenges to ICH treatment. However, the molecular level changes that occur in the brain after ICH remain largely unknown. Given that the aged population is the most rapidly growing population in America and possible increase in the incidence of ICH in the aging population, there is a need to conduct additional preclinical studies with old animal subjects for a better understanding of the role of aging in ICH pathology, which in turn would aid in the development of novel treatment strategies.

## Author Contributions

NW, FB, and SS-R: original draft preparation and writing. SS-R: conceptualization, editing, and funding acquisition. All authors contributed to the article and approved the submitted version.

## Conflict of Interest

The authors declare that the research was conducted in the absence of any commercial or financial relationships that could be construed as a potential conflict of interest.

## Publisher’s Note

All claims expressed in this article are solely those of the authors and do not necessarily represent those of their affiliated organizations, or those of the publisher, the editors and the reviewers. Any product that may be evaluated in this article, or claim that may be made by its manufacturer, is not guaranteed or endorsed by the publisher.

## Abbreviations

BMDM: brain-infiltrating monocyte-derived macrophage
CAA: cerebral amyloid angiopathy
CMB: cerebral microbleeds
CMH: cerebral microhemorrhages
ICH: intracerebral hemorrhage
IL-10: interleukin-10
IL-13: interleukin-13
IL-1 β: interleukin-1 β
IL-4: interleukin-4
IL-6: interleukin-6
IL-8: interleukin-8
IL-33: interleukin-33
LDL-C: LDL Cholestero
LPS: lipopolysaccharide
MAC: membrane attack complexes
MMP-9: matrix mettaloproteinase-9
MMP: matrix metalloproteinase
TGF- β: transforming growth factor beta
TLR4: toll like receptor 4
TNF- α: tumor necrosis factor alpha.

## References

[B1] Akoudad S. Portegies M. L. Koudstaal P. J. Hofman A. van der Lugt A. Ikram M. A. (2015). Cerebral Microbleeds Are Associated With an Increased Risk of Stroke: the Rotterdam Study. *Circulation* 132 509–516. 10.1161/CIRCULATIONAHA.115.016261 26137955

[B2] Albright J. M. Dunn R. C. Shults J. A. Boe D. M. Afshar M. Kovacs E. J. (2016). Advanced Age Alters Monocyte and Macrophage Responses. *Antioxid. Redox. Signal.* 25 805–815. 10.1089/ars.2016.6691 27357201 PMC5107740

[B3] An S. J. Kim T. J. Yoon B. W. (2017). Epidemiology, Risk Factors, and Clinical Features of Intracerebral Hemorrhage: an Update. *J. Stroke* 19 3–10. 10.5853/jos.2016.00864 28178408 PMC5307940

[B4] Ariesen M. J. Claus S. P. Rinkel G. J. Algra A. (2003). Risk factors for intracerebral hemorrhage in the general population: a systematic review. *Stroke* 34 2060–2065. 10.1161/01.STR.0000080678.09344.8D 12843354

[B5] Aronowski J. Hall C. E. (2005). New horizons for primary intracerebral hemorrhage treatment: experience from preclinical studies. *Neurol. Res.* 27 268–279. 10.1179/016164105X25225 15845210

[B6] Asdaghi N. Butcher K. S. Hill M. D. (2012). Risks and benefits of thrombolysis in the elderly. *Int. J. Stroke* 7 142–149. 10.1111/j.1747-4949.2011.00744.x 22264367

[B7] Bagg S. Pombo A. P. Hopman W. (2002). Effect of age on functional outcomes after stroke rehabilitation. *Stroke* 33 179–185. 10.1161/hs0102.101224 11779908

[B8] Baltan S. Besancon E. F. Mbow B. Ye Z. Hamner M. A. Ransom B. R. (2008). White matter vulnerability to ischemic injury increases with age because of enhanced excitotoxicity. *J. Neurosci.* 28 1479–1489. 10.1523/JNEUROSCI.5137-07.2008 18256269 PMC6671573

[B9] Bao W. D. Zhou X. T. Zhou L. T. Wang F. Yin X. Lu Y. (2020). Targeting miR-124/Ferroportin signaling ameliorated neuronal cell death through inhibiting apoptosis and ferroptosis in aged intracerebral hemorrhage murine model. *Aging Cell* 19:e13235. 10.1111/acel.13235 33068460 PMC7681046

[B10] Batista A. Osorio R. Varela A. Guilherme P. Marreiros A. Pais S. (2021). Prediction of short-term prognosis in elderly patients with spontaneous intracerebral hemorrhage. *Eur. Geriatr. Med.* 12 1267–1273. 10.1007/s41999-021-00529-w 34156657

[B11] Bonsack Ft Alleyne C. H. Jr. (2016). Sukumari-Ramesh S: augmented expression of TSPO after intracerebral hemorrhage: a role in inflammation? *J. Neuroinflamm.* 13:151. 10.1186/s12974-016-0619-2 27315802 PMC4912814

[B12] Broderick J. P. Brott T. Tomsick T. Miller R. Huster G. (1993). Intracerebral hemorrhage more than twice as common as subarachnoid hemorrhage. *J. Neurosurg.* 78 188–191. 10.3171/jns.1993.78.2.0188 8421201

[B13] Buford T. W. (2016). Hypertension and aging. *Ageing Res. Rev.* 26 96–111. 26835847 10.1016/j.arr.2016.01.007 PMC4768730

[B14] Candlish M. Hefendehl J. K. (2021). Microglia Phenotypes Converge in Aging and Neurodegenerative Disease. *Front. Neurol.* 12:660720. 10.3389/fneur.2021.660720 34025562 PMC8133315

[B15] Caro J. J. Huybrechts K. F. (1999). Stroke treatment economic model (STEM): predicting long-term costs from functional status. *Stroke* 30 2574–2579. 10.1161/01.str.30.12.2574 10582980

[B16] Carson M. J. Thrash J. C. Walter B. (2006). The cellular response in neuroinflammation: the role of leukocytes, microglia and astrocytes in neuronal death and survival. *Clin. Neurosci. Res.* 6 237–245. 10.1016/j.cnr.2006.09.004 19169437 PMC2630233

[B17] Chang C. F. Wan J. Li Q. Renfroe S. C. Heller N. M. Wang J. (2017). Alternative activation-skewed microglia/macrophages promote hematoma resolution in experimental intracerebral hemorrhage. *Neurobiol. Dis.* 103 54–69. 10.1016/j.nbd.2017.03.016 28365213 PMC5540140

[B18] Cheng Y. Qiao L. Jiang Z. Dong X. Feng H. Gui Q. (2020). Significant reduction in the LDL cholesterol increases the risk of intracerebral hemorrhage: a systematic review and meta-analysis of 33 randomized controlled trials. *Am. J. Transl. Res.* 12 463–477. 32194896 PMC7061840

[B19] Cheung R. T. Zou L. Y. (2003). Use of the original, modified, or new intracerebral hemorrhage score to predict mortality and morbidity after intracerebral hemorrhage. *Stroke* 34 1717–1722. 10.1161/01.STR.0000078657.22835.B9 12805488

[B20] Chou A. Krukowski K. Morganti J. M. Riparip L. K. Rosi S. (2018). Persistent Infiltration and Impaired Response of Peripherally-Derived Monocytes after Traumatic Brain Injury in the Aged Brain. *Int. J. Mol. Sci.* 19:1616. 10.3390/ijms19061616 29848996 PMC6032263

[B21] Conde J. R. Streit W. J. (2006). Microglia in the aging brain. *J. Neuropathol. Exp. Neurol.* 65 199–203. 16651881 10.1097/01.jnen.0000202887.22082.63

[B22] Craen A. Mangal R. Stead T. G. Ganti L. (2019). Gender Differences in Outcomes after Non-traumatic Intracerebral Hemorrhage. *Cureus* 11:e5818. 10.7759/cureus.5818 31737460 PMC6823069

[B23] Dai S. Hua Y. Keep R. F. Novakovic N. Fei Z. Xi G. (2019). Minocycline attenuates brain injury and iron overload after intracerebral hemorrhage in aged female rats. *Neurobiol. Dis.* 126 76–84. 10.1016/j.nbd.2018.06.001 29879529

[B24] Dasari R. Bonsack F. Sukumari-Ramesh S. (2021). Brain injury and repair after intracerebral hemorrhage: the role of microglia and brain-infiltrating macrophages. *Neurochem. Int.* 142:104923. 10.1016/j.neuint.2020.104923 33248206 PMC7818651

[B25] de Montgolfier O. Pouliot P. Gillis M. A. Ferland G. Lesage F. Thorin-Trescases N. (2019). Systolic hypertension-induced neurovascular unit disruption magnifies vascular cognitive impairment in middle-age atherosclerotic LDLr(-/-):hApoB(+/+) mice. *Geroscience* 41 511–532. 10.1007/s11357-019-00070-6 31093829 PMC6885084

[B26] Deleidi M. Jaggle M. Rubino G. (2015). Immune aging, dysmetabolism, and inflammation in neurological diseases. *Front. Neurosci.* 9:172. 10.3389/fnins.2015.00172 26089771 PMC4453474

[B27] Derraz I. Cagnazzo F. Gaillard N. Morganti R. Dargazanli C. Ahmed R. (2021). Microbleeds, Cerebral Hemorrhage, and Functional Outcome After Endovascular Thrombectomy. *Neurology* 96 e1724–e1731. 10.1212/WNL.0000000000011566 33495380

[B28] Dickson D. W. Crystal H. A. Mattiace L. A. Masur D. M. Blau A. D. Davies P. (1992). Identification of normal and pathological aging in prospectively studied nondemented elderly humans. *Neurobiol. Aging* 13 179–189. 10.1016/0197-4580(92)90027-u 1311804

[B29] Donnellan C. Werring D. (2020). Cognitive impairment before and after intracerebral haemorrhage: a systematic review. *Neurol. Sci.* 41 509–527. 10.1007/s10072-019-04150-5 31802344

[B30] Ducruet A. F. Zacharia B. E. Hickman Z. L. Grobelny B. T. Yeh M. L. Sosunov S. A. (2009). The complement cascade as a therapeutic target in intracerebral hemorrhage. *Exp. Neurol.* 219 398–403. 10.1016/j.expneurol.2009.07.018 19632224 PMC3731062

[B31] Fang H. Y. Ko W. J. Lin C. Y. (2007). Inducible heat shock protein 70, interleukin-18, and tumor necrosis factor alpha correlate with outcomes in spontaneous intracerebral hemorrhage. *J. Clin. Neurosci.* 14 435–441. 10.1016/j.jocn.2005.12.022 17336530

[B32] Feigin V. L. Lawes C. M. Bennett D. A. Barker-Collo S. L. Parag V. (2009). Worldwide stroke incidence and early case fatality reported in 56 population-based studies: a systematic review. *Lancet Neurol.* 8 355–369. 10.1016/S1474-4422(09)70025-0 19233729

[B33] Feldmann E. Broderick J. P. Kernan W. N. Viscoli C. M. Brass L. M. Brott T. (2005). Major risk factors for intracerebral hemorrhage in the young are modifiable. *Stroke* 36 1881–1885. 10.1161/01.STR.0000177480.62341.6b 16081867

[B34] Fenn A. M. Henry C. J. Huang Y. Dugan A. Godbout J. P. (2012). Lipopolysaccharide-induced interleukin (IL)-4 receptor-alpha expression and corresponding sensitivity to the M2 promoting effects of IL-4 are impaired in microglia of aged mice. *Brain Behav. Immun.* 26 766–777. 10.1016/j.bbi.2011.10.003 22024136 PMC3288757

[B35] Flaherty M. L. Woo D. Haverbusch M. Sekar P. Khoury J. Sauerbeck L. (2005). Racial variations in location and risk of intracerebral hemorrhage. *Stroke* 36 934–937. 10.1161/01.STR.0000160756.72109.95 15790947

[B36] Foreman K. E. Glovsky M. M. Warner R. L. Horvath S. J. Ward P. A. (1996). Comparative effect of C3a and C5a on adhesion molecule expression on neutrophils and endothelial cells. *Inflammation* 20 1–9. 10.1007/BF01487740 8926043

[B37] Frasca D. Blomberg B. B. (2016). Inflammaging decreases adaptive and innate immune responses in mice and humans. *Biogerontology* 17 7–19. 10.1007/s10522-015-9578-8 25921609 PMC4626429

[B38] Fujii Y. Tanaka R. Takeuchi S. Koike T. Minakawa T. Sasaki O. (1994). Hematoma enlargement in spontaneous intracerebral hemorrhage. *J. Neurosurg.* 80 51–57. 10.3171/jns.1994.80.1.0051 8271022

[B39] Gabuzda D. Yankner B. A. (2013). Physiology: inflammation links ageing to the brain. *Nature* 497 197–198. 10.1038/nature12100 23636321 PMC3860882

[B40] Ginaldi L. De Martinis M. D’Ostilio A. Marini L. Loreto M. F. Quaglino D. (1999). Immunological changes in the elderly. *Aging* 11 281–286. 10.1007/bf03339801 10631876

[B41] Godbout J. P. Chen J. Abraham J. Richwine A. F. Berg B. M. Kelley K. W. (2005). Exaggerated neuroinflammation and sickness behavior in aged mice following activation of the peripheral innate immune system. *FASEB J.* 19 1329–1331. 10.1096/fj.05-3776fje 15919760

[B42] Gong Y. Hua Y. Keep R. F. Hoff J. T. Xi G. (2004). Intracerebral hemorrhage: effects of aging on brain edema and neurological deficits. *Stroke* 35 2571–2575. 10.1161/01.STR.0000145485.67827.d0 15472083

[B43] Gong Y. Xi G. H. Keep R. F. Hoff J. T. Hua Y. (2005). Aging enhances intracerebral hemorrhage-induced brain injury in rats. *Acta Neurochir. Suppl.* 95 425–427. 10.1007/3-211-32318-x_87 16463895

[B44] Gong Y. Xi G. Wan S. Gu Y. Keep R. F. Hua Y. (2008). Effects of aging on complement activation and neutrophil infiltration after intracerebral hemorrhage. *Acta Neurochir. Suppl.* 105 67–70. 10.1007/978-3-211-09469-3_14 19066085

[B45] Gonzalez-Perez A. Gaist D. Wallander M. A. McFeat G. Garcia-Rodriguez L. A. (2013). Mortality after hemorrhagic stroke: data from general practice (The Health Improvement Network). *Neurology* 81 559–565. 10.1212/WNL.0b013e31829e6eff 23843467

[B46] Greenberg S. M. Eng J. A. Ning M. Smith E. E. Rosand J. (2004). Hemorrhage burden predicts recurrent intracerebral hemorrhage after lobar hemorrhage. *Stroke* 35 1415–1420. 10.1161/01.STR.0000126807.69758.0e 15073385

[B47] Hartman R. Lekic T. Rojas H. Tang J. Zhang J. H. (2009). Assessing functional outcomes following intracerebral hemorrhage in rats. *Brain Res.* 1280 148–157. 10.1016/j.brainres.2009.05.038 19464275 PMC6918942

[B48] Hemphill J. C. III Bonovich D. C. Besmertis L. Manley G. T. Johnston S. C. (2001). The ICH score: a simple, reliable grading scale for intracerebral hemorrhage. *Stroke* 32 891–897. 10.1161/01.str.32.4.891 11283388

[B49] Henry C. J. Huang Y. Wynne A. M. Godbout J. P. (2009). Peripheral lipopolysaccharide (LPS) challenge promotes microglial hyperactivity in aged mice that is associated with exaggerated induction of both pro-inflammatory IL-1beta and anti-inflammatory IL-10 cytokines. *Brain Behav. Immun.* 23 309–317. 10.1016/j.bbi.2008.09.002 18814846 PMC2692986

[B50] Hickenbottom S. L. Grotta J. C. Strong R. Denner L. A. Aronowski J. (1999). Nuclear factor-kappaB and cell death after experimental intracerebral hemorrhage in rats. *Stroke* 30 2472–2477. 10.1161/01.str.30.11.2472 10548686

[B51] Hoffman W. E. Pelligrino D. Miletich D. J. Albrecht R. F. (1985). Brain metabolic changes in young vs aged rats during hypoxia. *Stroke* 16 860–863. 10.1161/01.str.16.5.860 4049450

[B52] Hua Y. Xi G. Keep R. F. Hoff J. T. (2000). Complement activation in the brain after experimental intracerebral hemorrhage. *J. Neurosurg.* 92 1016–1022. 10.3171/jns.2000.92.6.1016 10839264

[B53] Huang J. Yang G. Xiong X. Wang M. Yuan J. Zhang Q. (2020). Age-related CCL12 Aggravates Intracerebral Hemorrhage-induced Brain Injury via Recruitment of Macrophages and T Lymphocytes. *Aging Dis.* 11 1103–1115. 10.14336/AD.2019.1229 33014526 PMC7505273

[B54] Inoue Y. Miyashita F. Minematsu K. Toyoda K. (2018). Clinical Characteristics and Outcomes of Intracerebral Hemorrhage in Very Elderly. *J. Stroke Cerebrovasc. Dis.* 27 97–102. 10.1016/j.jstrokecerebrovasdis.2017.08.006 28893575

[B55] Itoh Y. Yamada M. Hayakawa M. Otomo E. Miyatake T. (1993). Cerebral amyloid angiopathy: a significant cause of cerebellar as well as lobar cerebral hemorrhage in the elderly. *J. Neurol. Sci.* 116 135–141. 10.1016/0022-510x(93)90317-r 8336159

[B56] Jabbarli R. Reinhard M. Roelz R. Shah M. Niesen W. D. Kaier K. (2016). Intracerebral Hematoma Due to Aneurysm Rupture: are There Risk Factors Beyond Aneurysm Location? *Neurosurgery* 78 813–820. 10.1227/NEU.0000000000001136 26619334

[B57] Jackson C. A. Sudlow C. L. (2006). Is hypertension a more frequent risk factor for deep than for lobar supratentorial intracerebral haemorrhage? *J. Neurol. Neurosurg. Psychiatry* 77 1244–1252. 10.1136/jnnp.2006.089292 16690694 PMC2077396

[B58] Judge C. Ruttledge S. Costello M. Murphy R. Loughlin E. Alvarez-Iglesias A. (2019). Lipid Lowering Therapy, Low-Density Lipoprotein Level and Risk of Intracerebral Hemorrhage - A Meta-Analysis. *J. Stroke Cerebrovasc. Dis.* 28 1703–1709. 10.1016/j.jstrokecerebrovasdis.2019.02.018 30878368

[B59] Koellhoffer E. C. McCullough L. D. Ritzel R. M. (2017). Old Maids: Aging and Its Impact on Microglia Function. *Int. J. Mol. Sci.* 18:769. 10.3390/ijms18040769 28379162 PMC5412353

[B60] Lan X. Han X. Li Q. Li Q. Gao Y. Cheng T. (2017). Pinocembrin protects hemorrhagic brain primarily by inhibiting toll-like receptor 4 and reducing M1 phenotype microglia. *Brain Behav. Immun.* 61 326–339. 10.1016/j.bbi.2016.12.012 28007523 PMC5453178

[B61] Lee C. K. Klopp R. G. Weindruch R. Prolla T. A. (1999). Gene expression profile of aging and its retardation by caloric restriction. *Science* 285 1390–1393. 10.1126/science.285.5432.1390 10464095

[B62] Lee C. Z. Xue Z. Zhu Y. Yang G. Y. Young W. L. (2007). Matrix metalloproteinase-9 inhibition attenuates vascular endothelial growth factor-induced intracerebral hemorrhage. *Stroke* 38 2563–2568. 10.1161/STROKEAHA.106.481515 17673717

[B63] Lee J. C. Cho G. S. Choi B. O. Kim H. C. Kim W. K. (2009). Aging exacerbates intracerebral hemorrhage-induced brain injury. *J. Neurotrauma* 26 1567–1576. 10.1089/neu.2008.0630 19473060

[B64] Lee J. M. Yin K. J. Hsin I. Chen S. Fryer J. D. Holtzman D. M. (2003). Matrix metalloproteinase-9 and spontaneous hemorrhage in an animal model of cerebral amyloid angiopathy. *Ann. Neurol.* 54 379–382. 10.1002/ana.10671 12953271

[B65] Leira R. Davalos A. Silva Y. Gil-Peralta A. Tejada J. Garcia M. (2004). Stroke Project CDGotSNS: early neurologic deterioration in intracerebral hemorrhage: predictors and associated factors. *Neurology* 63 461–467. 10.1212/01.wnl.0000133204.81153.ac 15304576

[B66] Li J. Xiao L. He D. Luo Y. Sun H. (2021). Mechanism of White Matter Injury and Promising Therapeutic Strategies of MSCs After Intracerebral Hemorrhage. *Front. Aging Neurosci.* 13:632054. 10.3389/fnagi.2021.632054 33927608 PMC8078548

[B67] Lin S. Yin Q. Zhong Q. Lv F. L. Zhou Y. Li J. Q. (2012). Heme activates TLR4-mediated inflammatory injury via MyD88/TRIF signaling pathway in intracerebral hemorrhage. *J. Neuroinflammation* 9:46. 10.1186/1742-2094-9-46 22394415 PMC3344687

[B68] Love S. Nicoll J. A. Hughes A. Wilcock G. K. (2003). APOE and cerebral amyloid angiopathy in the elderly. *Neuroreport* 14 1535–1536. 10.1097/00001756-200308060-00027 12960780

[B69] Ma C. Gurol M. E. Huang Z. Lichtenstein A. H. Wang X. Wang Y. (2019). Low-density lipoprotein cholesterol and risk of intracerebral hemorrhage: a prospective study. *Neurology* 93 e445–e457. 31266905 10.1212/WNL.0000000000007853 PMC6693427

[B70] MacLellan C. L. Langdon K. D. Churchill K. P. Granter-Button S. Corbett D. (2009). Assessing cognitive function after intracerebral hemorrhage in rats. *Behav. Brain Res.* 198 321–328. 10.1016/j.bbr.2008.11.004 19041895

[B71] Matsukawa H. Shinoda M. Fujii M. Takahashi O. Yamamoto D. Murakata A. (2012). Factors associated with lobar vs. non-lobar intracerebral hemorrhage. *Acta Neurol. Scand.* 126 116–121. 10.1111/j.1600-0404.2011.01615.x 22067041

[B72] Mehndiratta P. Manjila S. Ostergard T. Eisele S. Cohen M. L. Sila C. (2012). Cerebral amyloid angiopathy-associated intracerebral hemorrhage: pathology and management. *Neurosurg. Focus* 32:E7. 10.3171/2012.1.FOCUS11370 22463117

[B73] Melton L. M. Keith A. B. Davis S. Oakley A. E. Edwardson J. A. Morris C. M. (2003). Chronic glial activation, neurodegeneration, and APP immunoreactive deposits following acute administration of double-stranded RNA. *Glia* 44 1–12. 10.1002/glia.10276 12951652

[B74] Miao Y. Zhang Z.-X. Feng X. Sun W.-M. (2021). IL-33 as a Novel Serum Prognostic Marker of Intracerebral Hemorrhage. *Oxid. Med. Cell. Long.* 2021:5597790. 10.1155/2021/5597790 33854693 PMC8019392

[B75] Morotti A. Goldstein J. N. (2016). Diagnosis and Management of Acute Intracerebral Hemorrhage. *Emerg. Med. Clin. North Am.* 34 883–899. 10.1016/j.emc.2016.06.010 27741993 PMC5089075

[B76] Mozaffarian D. Benjamin E. J. Go A. S. Arnett D. K. Blaha M. J. Cushman M. (2015). Heart Disease and Stroke Statistics-2016 Update: a Report From the American Heart Association. *Circulation* 133 e38–360. 26673558 10.1161/CIR.0000000000000350

[B77] Nakamura T. Keep R. F. Hua Y. Schallert T. Hoff J. T. Xi G. (2003). Deferoxamine-induced attenuation of brain edema and neurological deficits in a rat model of intracerebral hemorrhage. *Neurosurg. Focus* 15:EC4. 10.3171/foc.2003.15.4.10 15344903

[B78] Nakamura T. Xi G. Park J. W. Hua Y. Hoff J. T. Keep R. F. (2005). Holo-transferrin and thrombin can interact to cause brain damage. *Stroke* 36 348–352. 10.1161/01.STR.0000153044.60858.1b 15637325

[B79] Nakanishi H. (2003). Microglial functions and proteases. *Mol. Neurobiol.* 27 163–176. 10.1385/MN:27:2:163 12777686

[B80] Ni W. Mao S. Xi G. Keep R. F. Hua Y. (2016). Role of Erythrocyte CD47 in Intracerebral Hematoma Clearance. *Stroke* 47 505–511. 10.1161/STROKEAHA.115.010920 26732568 PMC4729651

[B81] Ni W. Okauchi M. Hatakeyama T. Gu Y. Keep R. F. Xi G. (2015). Deferoxamine reduces intracerebral hemorrhage-induced white matter damage in aged rats. *Exp. Neurol.* 272 128–134. 10.1016/j.expneurol.2015.02.035 25749188 PMC4668331

[B82] Nicoll J. A. Burnett C. Love S. Graham D. I. Dewar D. Ironside J. W. (1997). High frequency of apolipoprotein E epsilon 2 allele in hemorrhage due to cerebral amyloid angiopathy. *Ann. Neurol.* 41 716–721. 10.1002/ana.410410607 9189032

[B83] Nyul-Toth A. Fulop G. A. Tarantini S. Kiss T. Ahire C. Faakye J. A. (2022). Cerebral venous congestion exacerbates cerebral microhemorrhages in mice. *Geroscience* Epub online ahead of print. 10.1007/s11357-021-00504-0 34989944 PMC9135950

[B84] Ogura K. Ogawa M. Yoshida M. (1994). Effects of ageing on microglia in the normal rat brain: immunohistochemical observations. *Neuroreport* 5 1224–1226. 10.1097/00001756-199406020-00016 7919169

[B85] Orbach A. Zelig O. Yedgar S. Barshtein G. (2017). Biophysical and Biochemical Markers of Red Blood Cell Fragility. *Transfus Med. Hemother.* 44 183–187. 10.1159/000452106 28626369 PMC5473068

[B86] Orre M. Kamphuis W. Osborn L. M. Jansen A. H. P. Kooijman L. Bossers K. (2014). Isolation of glia from Alzheimer’s mice reveals inflammation and dysfunction. *Neurobiol. Aging* 35 2746–2760. 10.1016/j.neurobiolaging.2014.06.004 25002035

[B87] Ovbiagele B. Goldstein L. B. Higashida R. T. Howard V. J. Johnston S. C. Khavjou O. A. (2013). Forecasting the future of stroke in the United States: a policy statement from the American Heart Association and American Stroke Association. *Stroke* 44 2361–2375. 10.1161/STR.0b013e31829734f2 23697546

[B88] Pan J. Ma N. Yu B. Zhang W. Wan J. (2020). Transcriptomic profiling of microglia and astrocytes throughout aging. *J. Neuroinflammation* 17:97. 10.1186/s12974-020-01774-9 32238175 PMC7115095

[B89] Peters A. Josephson K. Vincent S. L. (1991). Effects of aging on the neuroglial cells and pericytes within area 17 of the rhesus monkey cerebral cortex. *Anat. Rec.* 229 384–398. 10.1002/ar.1092290311 2024779

[B90] Platt N. da Silva R. P. Gordon S. (1998). Recognizing death: the phagocytosis of apoptotic cells. *Trends Cell Biol.* 8 365–372. 10.1016/s0962-8924(98)01329-4 9728398

[B91] Poon M. T. Fonville A. F. Al-Shahi Salman R. (2014). Long-term prognosis after intracerebral haemorrhage: systematic review and meta-analysis. *J. Neurol. Neurosurg. Psychiatry* 85 660–667. 10.1136/jnnp-2013-306476 24262916

[B92] Qin L. Liu Y. Hong J. S. Crews F. T. (2013). NADPH oxidase and aging drive microglial activation, oxidative stress, and dopaminergic neurodegeneration following systemic LPS administration. *Glia* 61 855–868. 10.1002/glia.22479 23536230 PMC3631289

[B93] Qureshi A. I. Mendelow A. D. Hanley D. F. (2009). Intracerebral haemorrhage. *Lancet* 373 1632–1644. 19427958 10.1016/S0140-6736(09)60371-8 PMC3138486

[B94] Qureshi A. I. Suri M. F. Nasar A. Kirmani J. F. Ezzeddine M. A. Divani A. A. (2007). Changes in cost and outcome among US patients with stroke hospitalized in 1990 to 1991 and those hospitalized in 2000 to 2001. *Stroke* 38 2180–2184. 10.1161/STROKEAHA.106.467506 17525400

[B95] Rahpeymai Y. Hietala M. A. Wilhelmsson U. Fotheringham A. Davies I. Nilsson A. K. (2006). Complement: a novel factor in basal and ischemia-induced neurogenesis. *EMBO J.* 25 1364–1374. 10.1038/sj.emboj.7601004 16498410 PMC1422160

[B96] Ramos P. Santos A. Pinto N. R. Mendes R. Magalhaes T. Almeida A. (2014). Iron levels in the human brain: a post-mortem study of anatomical region differences and age-related changes. *J. Trace. Elem. Med. Biol.* 28 13–17. 10.1016/j.jtemb.2013.08.001 24075790

[B97] Rawji K. S. Mishra M. K. Michaels N. J. Rivest S. Stys P. K. Yong V. W. (2016). Immunosenescence of microglia and macrophages: impact on the ageing central nervous system. *Brain* 139 653–661. 10.1093/brain/awv395 26912633 PMC5839598

[B98] Reed S. D. Blough D. K. Meyer K. Jarvik J. G. (2001). Inpatient costs, length of stay, and mortality for cerebrovascular events in community hospitals. *Neurology* 57 305–314. 10.1212/wnl.57.2.305 11468317

[B99] Sacco S. Marini C. Toni D. Olivieri L. Carolei A. (2009). Incidence and 10-year survival of intracerebral hemorrhage in a population-based registry. *Stroke* 40 394–399. 10.1161/STROKEAHA.108.523209 19038914

[B100] Shahidehpour R. K. Higdon R. E. Crawford N. G. Neltner J. H. Ighodaro E. T. Patel E. (2021). Dystrophic microglia are associated with neurodegenerative disease and not healthy aging in the human brain. *Neurobiol. Aging* 99 19–27. 10.1016/j.neurobiolaging.2020.12.003 33422891 PMC8293930

[B101] Shao A. Lin D. Wang L. Tu S. Lenahan C. Zhang J. (2020). Oxidative Stress at the Crossroads of Aging. Stroke and Depression. *Aging Dis.* 11 1537–1566. 10.14336/AD.2020.0225 33269106 PMC7673857

[B102] Sheffield L. G. Berman N. E. (1998). Microglial expression of MHC class II increases in normal aging of nonhuman primates. *Neurobiol. Aging* 19 47–55. 10.1016/s0197-4580(97)00168-1 9562503

[B103] Shiratori M. Tozaki-Saitoh H. Yoshitake M. Tsuda M. Inoue K. (2010). P2X7 receptor activation induces CXCL2 production in microglia through NFAT and PKC/MAPK pathways. *J. Neurochem.* 114 810–819. 10.1111/j.1471-4159.2010.06809.x 20477948

[B104] Sierra A. Gottfried-Blackmore A. C. McEwen B. S. Bulloch K. (2007). Microglia derived from aging mice exhibit an altered inflammatory profile. *Glia* 55 412–424. 10.1002/glia.20468 17203473

[B105] Silva Y. Leira R. Tejada J. Lainez J. M. Castillo J. Davalos A. (2005). Stroke Project CDGotSNS: molecular signatures of vascular injury are associated with early growth of intracerebral hemorrhage. *Stroke* 36 86–91. 10.1161/01.STR.0000149615.51204.0b 15550687

[B106] Sims G. P. Rowe D. C. Rietdijk S. T. Herbst R. Coyle A. J. (2010). HMGB1 and RAGE in inflammation and cancer. *Annu. Rev. Immunol.* 28 367–388. 10.1146/annurev.immunol.021908.132603 20192808

[B107] Smith E. E. Gurol M. E. Eng J. A. Engel C. R. Nguyen T. N. Rosand J. (2004). White matter lesions, cognition, and recurrent hemorrhage in lobar intracerebral hemorrhage. *Neurology* 63 1606–1612. 10.1212/01.wnl.0000142966.22886.20 15534243

[B108] Spittau B. (2017). Aging Microglia-Phenotypes, Functions and Implications for Age-Related Neurodegenerative Diseases. *Front. Aging Neurosci.* 9:194. 10.3389/fnagi.2017.00194 28659790 PMC5469878

[B109] Starossom S. C. Mascanfroni I. D. Imitola J. Cao L. Raddassi K. Hernandez S. F. (2012). Galectin-1 deactivates classically activated microglia and protects from inflammation-induced neurodegeneration. *Immunity* 37 249–263. 10.1016/j.immuni.2012.05.023 22884314 PMC3428471

[B110] Stein M. Misselwitz B. Hamann G. F. Scharbrodt W. Schummer D. I. Oertel M. F. (2012). Intracerebral hemorrhage in the very old: future demographic trends of an aging population. *Stroke* 43 1126–1128. 10.1161/STROKEAHA.111.644716 22282880

[B111] Sudlow C. Martinez Gonzalez N. A. Kim J. Clark C. (2006). Does apolipoprotein E genotype influence the risk of ischemic stroke, intracerebral hemorrhage, or subarachnoid hemorrhage? Systematic review and meta-analyses of 31 studies among 5961 cases and 17,965 controls. *Stroke* 37 364–370. 10.1161/01.STR.0000199065.12908.62 16385096 PMC2577180

[B112] Sun Z. (2015). Aging, arterial stiffness, and hypertension. *Hypertension* 65 252–256. 10.1161/hypertensionaha.114.03617 25368028 PMC4288978

[B113] Taetzsch T. Levesque S. McGraw C. Brookins S. Luqa R. Bonini M. G. (2015). Redox regulation of NF-kappaB p50 and M1 polarization in microglia. *Glia* 63 423–440. 10.1002/glia.22762 25331559 PMC4322433

[B114] Talha K. A. Selina F. Nasir M. Kausar A. Islam T. Perveen R. A. (2020). Systematic Review on Apolipoprotein E: a Strong Genetic Cause of Hemorrhagic Stroke. *Mymensingh Med. J.* 29 1026–1032. 33116113

[B115] Tang J. Liu J. Zhou C. Alexander J. S. Nanda A. Granger D. N. (2004). Mmp-9 deficiency enhances collagenase-induced intracerebral hemorrhage and brain injury in mutant mice. *J. Cereb. Blood Flow Metab.* 24 1133–1145. 10.1097/01.WCB.0000135593.05952.DE 15529013

[B116] Tao C. Hu X. Li H. You C. (2017). White Matter Injury after Intracerebral Hemorrhage: Pathophysiology and Therapeutic Strategies. *Front. Hum. Neurosci.* 11:422. 10.3389/fnhum.2017.00422 28890692 PMC5575148

[B117] Tao C. Zhang R. Hu X. Song L. Wang C. Gao F. (2016). A Novel Brainstem Hemorrhage Model by Autologous Blood Infusion in Rat: white Matter Injury, Magnetic Resonance Imaging, and Neurobehavioral Features. *J. Stroke Cerebrovasc. Dis.* 25 1102–1109. 10.1016/j.jstrokecerebrovasdis.2016.01.025 26888564

[B118] Tarantini S. Valcarcel-Ares N. M. Yabluchanskiy A. Springo Z. Fulop G. A. Ashpole N. (2017). Insulin-like growth factor 1 deficiency exacerbates hypertension-induced cerebral microhemorrhages in mice, mimicking the aging phenotype. *Aging Cell* 16 469–479. 10.1111/acel.12583 28295976 PMC5418199

[B119] Tarantini S. Yabluchanskiy A. Lindsey M. L. Csiszar A. Ungvari Z. (2021). Effect of genetic depletion of MMP-9 on neurological manifestations of hypertension-induced intracerebral hemorrhages in aged mice. *Geroscience* 43 2611–2619. 10.1007/s11357-021-00402-5 34415518 PMC8599521

[B120] Taylor T. N. Davis P. H. Torner J. C. Holmes J. Meyer J. W. Jacobson M. F. (1996). Lifetime cost of stroke in the United States. *Stroke* 27 1459–1466. 10.1161/01.str.27.9.1459 8784113

[B121] Tessier P. A. Naccache P. H. Clark-Lewis I. Gladue R. P. Neote K. S. McColl S. R. (1997). Chemokine networks in vivo: involvement of C-X-C and C-C chemokines in neutrophil extravasation in vivo in response to TNF-alpha. *J. Immunol.* 159 3595–3602. 9317159

[B122] Tichauer J. E. Flores B. Soler B. Eugenin-von Bernhardi L. Ramirez G. von Bernhardi R. (2014). Age-dependent changes on TGFbeta1 Smad3 pathway modify the pattern of microglial cell activation. *Brain Behav. Immun.* 37 187–196. 10.1016/j.bbi.2013.12.018 24380849 PMC3951654

[B123] Toth P. Tarantini S. Springo Z. Tucsek Z. Gautam T. Giles C. B. (2015). Aging exacerbates hypertension-induced cerebral microhemorrhages in mice: role of resveratrol treatment in vasoprotection. *Aging Cell.* 14 400–408. 10.1111/acel.12315 25677910 PMC4406669

[B124] Tsushima Y. Aoki J. Endo K. (2003). Brain microhemorrhages detected on T2*-weighted gradient-echo MR images. *AJNR Am. J. Neuroradiol.* 24 88–96. 12533332 PMC8148967

[B125] Umeano O. Phillips-Bute B. Hailey C. E. Sun W. Gray M. C. Roulhac-Wilson B. (2013). Gender and age interact to affect early outcome after intracerebral hemorrhage. *PLoS One* 8:e81664. 10.1371/journal.pone.0081664 24312335 PMC3842307

[B126] van Asch C. J. Luitse M. J. Rinkel G. J. van der Tweel I. Algra A. Klijn C. J. (2010a). Incidence, case fatality, and functional outcome of intracerebral haemorrhage over time, according to age, sex, and ethnic origin: a systematic review and meta-analysis. *Lancet Neurol.* 9 167–176. 10.1016/S1474-4422(09)70340-0 20056489

[B127] van Asch C. J. Oudendijk J. F. Rinkel G. J. Klijn C. J. (2010b). Early intracerebral hematoma expansion after aneurysmal rupture. *Stroke* 41 2592–2595. 10.1161/STROKEAHA.110.589291 20947853

[B128] Vernooij M. W. van der Lugt A. Ikram M. A. Wielopolski P. A. Niessen W. J. Hofman A. (2008). Prevalence and risk factors of cerebral microbleeds: the Rotterdam Scan Study. *Neurology* 70 1208–1214. 10.1212/01.wnl.0000307750.41970.d9 18378884

[B129] Viswanathan A. Greenberg S. M. (2011). Cerebral amyloid angiopathy in the elderly. *Ann. Neurol.* 70 871–880. 22190361 10.1002/ana.22516 PMC4004372

[B130] Wan S. Cheng Y. Jin H. Guo D. Hua Y. Keep R. F. (2016). Microglia Activation and Polarization After Intracerebral Hemorrhage in Mice: the Role of Protease-Activated Receptor-1. *Transl. Stroke Res.* 7 478–487. 10.1007/s12975-016-0472-8 27206851 PMC5065741

[B131] Wang J. Dore S. (2007). Inflammation after intracerebral hemorrhage. *J. Cereb. Blood Flow Metab* 27 894–908. 10.1038/sj.jcbfm.9600403 17033693

[B132] Wang Y. C. Wang P. F. Fang H. Chen J. Xiong X. Y. Yang Q. W. (2013). Toll-like receptor 4 antagonist attenuates intracerebral hemorrhage-induced brain injury. *Stroke* 44 2545–2552. 10.1161/STROKEAHA.113.001038 23839500

[B133] Ward R. J. Zucca F. A. Duyn J. H. Crichton R. R. Zecca L. (2014). The role of iron in brain ageing and neurodegenerative disorders. *Lancet Neurol.* 13 1045–1060. 10.1016/S1474-4422(14)70117-6 25231526 PMC5672917

[B134] Wasil T. Lichtman S. M. (2005). Treatment of elderly cancer patients with chemotherapy. *Cancer Invest.* 23 537–547. 10.1080/07357900500202770 16203662

[B135] Wasserman J. K. Yang H. Schlichter L. C. (2008). Glial responses, neuron death and lesion resolution after intracerebral hemorrhage in young vs. aged rats. *Eur. J. Neurosci.* 28 1316–1328. 10.1111/j.1460-9568.2008.06442.x 18973558

[B136] Winkler D. T. Bondolfi L. Herzig M. C. Jann L. Calhoun M. E. Wiederhold K. H. (2001). Spontaneous hemorrhagic stroke in a mouse model of cerebral amyloid angiopathy. *J. Neurosci.* 21 1619–1627. 10.1523/JNEUROSCI.21-05-01619.2001 11222652 PMC6762950

[B137] Wu J. Hua Y. Keep R. F. Nakamura T. Hoff J. T. Xi G. (2003). Iron and iron-handling proteins in the brain after intracerebral hemorrhage. *Stroke* 34 2964–2969. 10.1161/01.STR.0000103140.52838.45 14615611

[B138] Xi G. Hua Y. Keep R. F. Younger J. G. Hoff J. T. (2001). Systemic complement depletion diminishes perihematomal brain edema in rats. *Stroke* 32 162–167. 10.1161/01.str.32.1.162 11136932

[B139] Xi G. Hua Y. Keep R. F. Younger J. G. Hoff J. T. (2002a). Brain edema after intracerebral hemorrhage: the effects of systemic complement depletion. *Acta Neurochir. Suppl.* 81 253–256. 10.1007/978-3-7091-6738-0_66 12168319

[B140] Xi G. Keep R. F. Hoff J. T. (2002b). Pathophysiology of brain edema formation. *Neurosurg. Clin. North Am.* 13 371–383. 10.1016/s1042-3680(02)00007-4 12486926

[B141] Xiong L. Reijmer Y. D. Charidimou A. Cordonnier C. Viswanathan A. (2016). Intracerebral hemorrhage and cognitive impairment. *Biochim. Biophys. Acta* 1862 939–944. 26692171 10.1016/j.bbadis.2015.12.011

[B142] Xu X. Wang B. Ren C. Hu J. Greenberg D. A. Chen T. (2017). Age-related Impairment of Vascular Structure and Functions. *Aging Dis.* 8 590–610. 10.14336/AD.2017.0430 28966804 PMC5614324

[B143] Yang N. R. Kim J. H. Ahn J. H. Oh J. K. Chang I. B. Song J. H. (2020). Is nontraumatic intracerebral hemorrhage different between young and elderly patients? *Neurosurg. Rev.* 43 781–791. 10.1007/s10143-019-01120-5 31161443

[B144] Yang S. Nakamura T. Hua Y. Keep R. F. Younger J. G. He Y. (2006a). The role of complement C3 in intracerebral hemorrhage-induced brain injury. *J. Cereb. Blood Flow Metab.* 26 1490–1495. 10.1038/sj.jcbfm.9600305 16552422

[B145] Yang S. Nakamura T. Hua Y. Keep R. F. Younger J. G. Hoff J. T. (2006b). Intracerebral hemorrhage in complement C3-deficient mice. *Acta Neurochir. Suppl.* 96 227–231. 10.1007/3-211-30714-1_49 16671460

[B146] Yuan J. J. Zhang Q. Gong C. X. Wang F. X. Huang J. C. Yang G. Q. (2019). Young plasma ameliorates aging-related acute brain injury after intracerebral hemorrhage. *Biosci. Rep.* 39:BSR20190537. 10.1042/BSR20190537 31040201 PMC6522807

[B147] Zecca L. Stroppolo A. Gatti A. Tampellini D. Toscani M. Gallorini M. (2004). The role of iron and copper molecules in the neuronal vulnerability of locus coeruleus and substantia nigra during aging. *Proc. Natl. Acad. Sci. U. S. A.* 101 9843–9848. 10.1073/pnas.0403495101 15210960 PMC470762

[B148] Zhang Y. Yang Y. Zhang G. Z. Gao M. Ge G. Z. Wang Q. Q. (2016). Stereotactic Administration of Edaravone Ameliorates Collagenase-Induced Intracerebral Hemorrhage in Rat. *CNS Neurosci. Ther.* 22 824–835. 10.1111/cns.12584 27390192 PMC5095785

[B149] Zhao X. Zhang Y. Strong R. Zhang J. Grotta J. C. Aronowski J. (2007). Distinct patterns of intracerebral hemorrhage-induced alterations in NF-kappaB subunit, iNOS, and COX-2 expression. *J. Neurochem.* 101 652–663. 10.1111/j.1471-4159.2006.04414.x 17250675

[B150] Zia E. Engstrom G. Svensson P. J. Norrving B. Pessah-Rasmussen H. (2009). Three-year survival and stroke recurrence rates in patients with primary intracerebral hemorrhage. *Stroke* 40 3567–3573. 10.1161/STROKEAHA.109.556324 19729603

